# Dr. Coluthur Gopalan: Father of Indian Nutrition Science

**DOI:** 10.7759/cureus.67097

**Published:** 2024-08-17

**Authors:** Amar Mankar, Umesh Kawalkar, Abhay Gaidhane, Niraj P Shinde

**Affiliations:** 1 Community Medicine, Datta Meghe Institute of Higher Education and Research, Wardha, IND; 2 Community Medicine, Government Medical College, Akola, IND; 3 School of Epidemiology and Public Health, Jawaharlal Nehru Medical College, Wardha, IND; 4 Community Medicine, Datta Meghe Institute of Medical Sciences, Wardha, IND

**Keywords:** icmr, impact of malnutrition - mother and child nutrition, nutrition status, food and nutrition, historical vignette

## Abstract

Dr. Coluthur Gopalan, a towering figure in nutrition science in India, made seminal contributions that transformed public health and nutrition policy in the country. Born in Salem, Tamil Nadu, in 1918, Dr. Gopalan's illustrious academic journey began at the Christian College High School and Madras Medical College, where he earned his M.D. in General Medicine in 1943. The Bengal Famine of 1942 profoundly influenced his career, steering him towards nutrition research. Awarded the Nuffield Foundation Scholarship, he earned a Ph.D. in nutrition from the University of London in just 30 months. Upon his return to India, he joined the Nutrition Research Laboratories (NRL) in Coonoor in 1949, which later became the National Institute of Nutrition (NIN), where he significantly broadened the scope of nutrition research. Dr. Gopalan's work laid the foundation for pivotal national nutrition programs, such as the Integrated Child Development Services (ICDS) and the midday meal scheme for schoolchildren. His tenure as Director of NIN (1960-1974) and later as Director General of the Indian Council of Medical Research (ICMR) from 1974 to 1979 saw major advancements in addressing malnutrition and emerging issues like overnutrition. His dedication to improving women's and children's nutritional status left a lasting impact on public health in India. Dr. Gopalan's pioneering research on protein-calorie malnutrition, micronutrient deficiencies, and holistic approaches to nutritional problems provided critical insights and guided the national policies. As an institution builder, he transformed NIN into a premier research center and during his tenure established new research institutes at ICMR, fostering a robust framework for future research. His advocacy ensured that nutrition was prioritized in national development plans, leading to significant health improvements. Internationally recognized, Dr. Gopalan's contributions included efforts to improve global nutrition, earning him numerous accolades. His legacy, encapsulated in the Nutrition Foundation of India, and his several contributions continue to be a vital resource for nutritionists and policymakers, ensuring lasting benefits for future generations. Dr. Gopalan's compassionate personality, visionary leadership, and holistic approach have indelibly advanced the nutritional status and health of millions globally.

## Introduction and background

Early life and career

**Figure 1:**
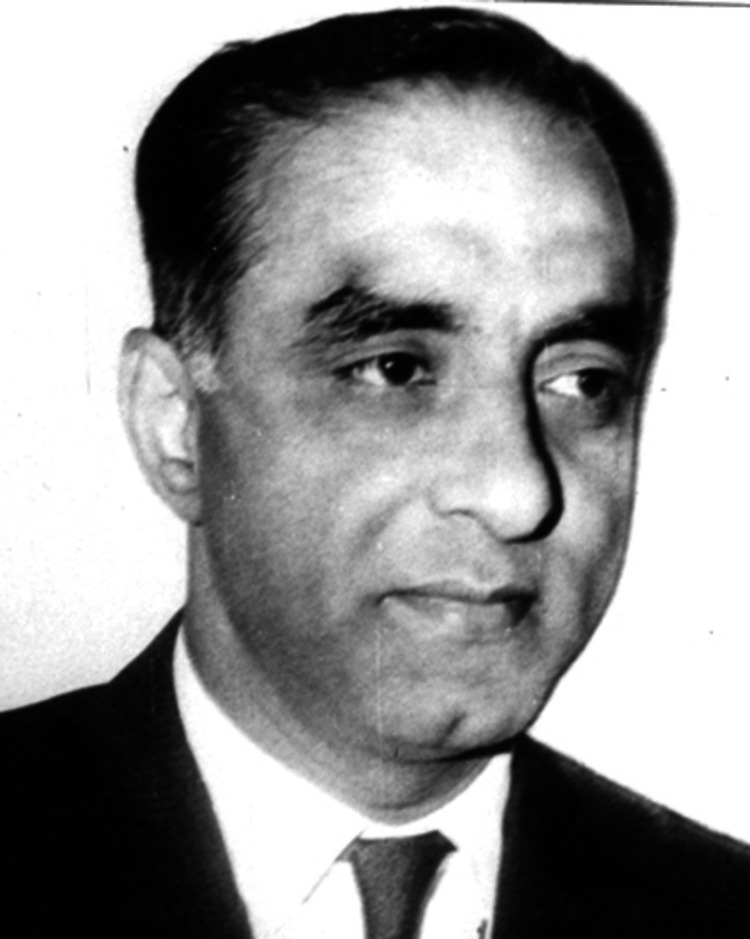
Dr. Coluthur Gopalan (1918-2019) Photo credit: Wikimedia Commons

Dr. Coluthur Gopalan (Figure 1) , born in Salem, Tamil Nadu, had a distinguished academic career. He began at Christian College High School and went on to earn his MD in General Medicine from Madras Medical College in 1943. The Bengal Famine of 1942 deeply impacted Dr. Gopalan, shifting his focus from clinical practice to nutrition research [1]. He became the first Indian to receive the Nuffield Foundation Scholarship, which enabled him to obtain a Ph.D. in nutrition from the University of London in just two and a half years [2].

Back in India, Dr. Gopalan joined the Nutrition Research Laboratories (NRL) in 1949, which in late 1950 was renamed the National Institute of Nutrition (NIN). During his tenure from late 1950 at NIN, he expanded nutrition research by integrating diverse disciplines like biochemistry, endocrinology, and toxicology. His dedication and leadership skills led him to become the Director of NIN (1960-1974) and later from 1974 to 1979 the Director General of the Indian Council of Medical Research (ICMR), where he greatly influenced national nutrition policies and programs [1].

## Review

Pioneering research in nutrition

Dr. Gopalan's research on protein-calorie malnutrition, specifically kwashiorkor and marasmus, was groundbreaking. He elucidated the clinical manifestations, biochemical changes, and underlying causes of these severe forms of malnutrition, which were prevalent among children in developing countries. His work highlighted the critical importance of adequate protein and calorie intake for preventing and treating these conditions, informing national and international nutrition guidelines [3]. Beyond his work on protein-energy malnutrition, Gopalan also made significant contributions to the understanding of pellagra, a niacin deficiency disease. His research helped to identify the dietary factors that contributed to pellagra and led to the development of effective prevention and treatment strategies. He also conducted important research on the role of nutrition in the prevention and treatment of other diseases, such as tuberculosis and leprosy. In addition to protein-calorie malnutrition, Dr. Gopalan conducted extensive research on micronutrient deficiencies, notably vitamin A deficiency, a leading cause of blindness in children. He established the link between undernutrition, vitamin A deficiency, and infections, paving the way for the National Programme for Prevention of Blindness. This program, which involved a massive dose of vitamin A supplementation, significantly reduced the incidence of keratomalacia and associated mortality rates [4]. Dr. Gopalan's research extended to other micronutrient deficiencies as well. His studies on iron deficiency anemia in pregnant women led to the implementation of iron and folic acid supplementation programs, which became a cornerstone of maternal health initiatives in India. He also investigated iodine deficiency disorders and advocated for universal salt iodization, a program that virtually eliminated iodine deficiency disorders such as goiter and cretinism in the country [5-7].

Recognizing the critical role of maternal nutrition in the health of both mother and child, Dr. Gopalan conducted extensive research on the nutritional needs of women during pregnancy and lactation. His work emphasized the importance of adequate nutrition for preventing anemia, promoting optimal fetal growth and development and ensuring adequate breast milk production. His advocacy for nutritional supplementation programs for pregnant and lactating women has had a lasting impact on maternal and child health in India [6]. Furthermore, Gopalan's work highlighted the importance of breastfeeding for infant health and development. He advocated for policies and programs to support breastfeeding, including the Baby Friendly Hospital Initiative. His research also contributed to a better understanding of the nutritional needs of infants and young children, informing the development of appropriate complementary feeding practices [7].

Development of national nutrition programs

Dr. Gopalan was not only a brilliant researcher but also a visionary leader who played a pivotal role in the development of several key health and nutrition research institutions in India. As the Director of NIN, he transformed the institute into a premier research center, expanding its scope to include various disciplines and establishing state-of-the-art laboratories and field units. Under his leadership, NIN conducted comprehensive research on the nutritive value of Indian foods, leading to the publication of dietary guidelines that were instrumental in formulating national nutrition policies [1]. During his tenure as Director General of the ICMR, Dr. Gopalan spearheaded the establishment of new research institutes focused on major communicable diseases such as leprosy, malaria, and filariasis. He also modernized the existing institutions and initiated the Talent Search Programme, which identified and nurtured young medical graduates interested in research. These efforts significantly strengthened India's medical research infrastructure and fostered a new generation of researchers [1]. Dr. Gopalan's research provided the scientific basis for several national nutrition programs that have had a far-reaching impact on public health in India. The Integrated Child Development Services (ICDS) scheme, launched in the 1970s, is a prime example [7]. This comprehensive program aimed to improve the health, nutrition, and development of children under six years of age, pregnant women, and lactating mothers by providing a package of services including supplementary nutrition, immunization, health check-ups, and referral services. The ICDS scheme has been instrumental in reducing malnutrition and improving child health outcomes in India [7]. Another notable program influenced by Dr. Gopalan's work is the mid-day meal scheme for schoolchildren. This program provides free lunches to children in government schools, addressing the issue of classroom hunger and improving school attendance. The mid-day meal scheme has not only improved the nutritional status of children but also contributed to better educational outcomes. Gopalan also played a key role in the development of the National Nutritional Anemia Prophylaxis Programme in 1970, which aimed to reduce the prevalence of anemia among women and children through iron and folic acid supplementation [7].

Dr. Gopalan was a tireless advocate for the integration of nutrition into public health policy. He emphasized the need for a multi-sectoral approach to address malnutrition, recognizing that it is a complex issue with roots in poverty, food insecurity, inadequate sanitation, and lack of access to healthcare. His advocacy efforts led to the inclusion of nutrition as a priority area in national development plans and policies adopted in 1993 by the Government of India. He also played a crucial role in establishing the National Nutrition Monitoring Bureau (NNMB) in 1972, which conducts regular surveys to assess the dietary intake, nutritional status, and health indicators of various population groups across India. The data collected by NNMB has been invaluable in identifying emerging nutritional challenges, monitoring the effectiveness of interventions, and formulating evidence-based policies [1]. Dr. Gopalan's contributions to nutrition science and public health extended beyond India's borders. He was actively involved in international nutrition research and policy, serving as the President of the International Union of Nutritional Sciences and the Chairman of the Regional Advisory Committee on Medical Research for the World Health Organization (WHO) from 1975 to 1980. His expertise and guidance were sought by developing countries around the world, and he played a key role in shaping global nutrition agendas. Dr. Gopalan played a pivotal role in establishing a network of Asian nutrition scientists and was instrumental in organizing the first Asian Congress of Nutrition, promoting its successors. His efforts contributed significantly to the creation of the Federation of Asian Nutrition Societies. He is recognized as a capable administrator and a visionary leader [1-3]. Dr. Gopalan's work was widely recognized and honored both nationally and internationally. He received numerous awards and accolades, including the Padma Shri (1970) and Padma Bhushan (2003), two of India's highest civilian awards. He was also elected a Fellow of the Royal Society (1987), a testament to his exceptional contributions to science [1]. Dr. Coluthur Gopalan's legacy is one of exceptional scientific achievement, visionary leadership, and unwavering commitment to improving the nutritional status and health of populations. His pioneering research, institutional development efforts, policy advocacy, and program implementation have left an enduring impact on the field of nutrition and public health [8]. The institutions he built, in late 1950 such as NIN and the research institutes at ICMR, continue to be at the forefront of nutrition research and training in India. The programs he initiated, such as the ICDS scheme (1970) and the mid-day meal scheme, continue to benefit millions of children and women across the country. The policies he influenced have shaped India's approach to nutrition and public health, contributing to significant improvements in the nutritional status of the population. Dr. Gopalan's seminal publication, *Nutritive Value of Indian Foods*, remains an essential reference for nutritionists, researchers, and policymakers [9]. His work on protein-calorie malnutrition, micronutrient deficiencies, and maternal nutrition continues to inform national and international nutrition guidelines [2].

## Conclusions

Dr. Coluthur Gopalan's contributions to the field of nutrition are immeasurable. His groundbreaking research, visionary leadership, and tireless advocacy have transformed the landscape of nutrition science and public health in India and beyond. His legacy at the NIN continues to inspire and guide future generations of researchers, policymakers, and practitioners, ensuring that his work continues to benefit millions of people around the world.
